# Synthesis of Polyazobenzenes Exhibiting Photoisomerization and Liquid Crystallinity

**DOI:** 10.3390/polym11020348

**Published:** 2019-02-17

**Authors:** Masashi Otaki, Reiji Kumai, Hajime Sagayama, Hiromasa Goto

**Affiliations:** 1 Department of Materials Science, Faculty of Pure and Applied Sciences, University of Tsukuba, Tsukuba, Ibaraki 305-8573, Japan; s1720420@s.tsukuba.ac.jp; 2 Photon Factory, Institute of Materials Structure Science, High Energy Accelerator Research Organization (KEK), Tsukuba, Ibaraki 305-0801, Japan; reiji.kumai@kek.jp (R.K.); hajime.sagayama@kek.jp (H.S.)

**Keywords:** Miyaura–Suzuki coupling, photo-isomerization, liquid crystal, synchrotron XRD.

## Abstract

While only a few studies have investigated the synthesis of main chain-type polyazobenzenes, they continue to draw an increasing amount of attention owing to their industrial applications in holography, dyes, and functional adhesives. In this study, dibromoazobenzene was prepared as a monomer for constructing azo-based π-conjugated polymers. Miyaura–Suzuki cross-coupling polymerization was conducted to develop copolymers containing an azobenzene unit as a photoisomerization block and a pyrimidine-based liquid crystal generator block. The prepared polymers exhibited thermotropic liquid crystallinity and underwent cis and trans photoisomerization upon irradiation with ultraviolet and visible light. Furthermore, the photoisomerization behavior was examined using optical absorption spectroscopy and synchrotron X-ray diffraction spectrometry.

## 1. Introduction

**Scheme 1:**
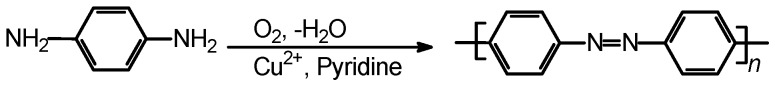
Synthesis of polyazobenzene [1].

In 1966, Bach reported the synthesis of main chain-type polyazobenzene based on Cu^2+^-promoted oxidative coupling (Scheme 1) [1] while developing π-conjugated polymers [2]. Thereafter, Berlin et al. prepared aromatic polymers containing an azo unit via the decomposition of bisdiazonium salts using Cu^+^ in 1961 [3]. Azoic molecules have been developed, which are used for developing dyes and pigments for coloration. Azoic dyes have been applied to liquid crystal alignment [4,5], holography [6], photo-refractivity [7], and reworkable adhesives [8]. Polymeric materials are also in demand owing to their film-forming properties. Recently, azo polymers exhibiting thermally-triggered degradation features have been integrated into drug delivery systems [9,10]. Side chain-type polyazobenzenes have also been used in various applications, including triggers to change nanostructures [11], biomimetic photoactuators [12], and light-responsive elastomers [13]. Despite progress in the research field of azobenzenes, studies on main chain-type polyazobenzenes exhibiting both liquid crystallinity and photoisomerization functions have not been reported to date.

**Figure 1:**
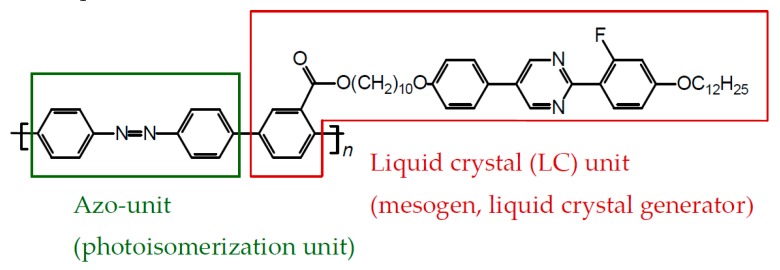
Functional blocks of the prepared polymer (poly1).

The synthesis of dibromoazobenzene using MnO_2_ as a catalyst for the reaction was studied in 1964 [14]. This simple and convenient method allows the synthesis of dibromoazobenzene, and subsequently, the corresponding main chain-type π-conjugated polymer containing azobenzene in the monomer repeat unit can be prepared via Pd(0)-catalyzed Miyaura–Suzuki polycondensation. In this paper, we introduce a method of synthesizing copolymers containing azobenzene and liquid crystal (LC) units to obtain photoisomerizable LC polymers that exhibit good film-forming properties. Figure 1 depicts the functional blocks of the prepared polymer (abbreviated as poly1). The azobenzene in the main chain (azo unit) provides the photoisomerization characteristics, whereas the LC unit exhibits the mesophase behavior.

## 2. Materials

[1,1′-bis(diphenylphosphino)ferrocene]dichloropalladium(II) complex/dichloromethane was purchased from Tokyo Chemical Industry (TCI, Tokyo, Japan). 4-[2-(4-Dodecyloxy-2-fluorophenyl)-pyrimidine-5-yl]-phenol, as a precursor of the liquid crystal molecules, was gifted to us by Midori Kagaku Co. (Midori Chemical, Tokyo, Japan). Bis(4,4,5,5-tetramethyl-[1,3]dioxolan-2-yl)borane was purchased from TCI. Fourier transform infrared (FT–IR) absorption spectroscopy measurements were performed using FT–IR 4600 (JASCO, Tokyo, Japan) based on the KBr method. Further, the molecular weights of the polymers were determined by gel permeation chromatography (GPC) with a MIXED-D HPLC column (Polymer Laboratories, Church Stretton, UK), a PU-980 HPLC pump (JASCO, Tokyo, Japan), and an MD-915 multi-wavelength detector (JASCO) using tetrahydrofuran (THF) as the solvent. All the instruments were calibrated based on the polystyrene standard. The ^1^H nuclear magnetic resonance (NMR) spectra were measured in CDCl_3_ using a JNM-ECS-400 NMR spectrometer (JEOL, Tokyo, Japan). Further, the chemical shifts were recorded in parts per million downfield from the internal standard tetramethylsilane (TMS). The absorption spectra were obtained using a V-630 spectrometer (JASCO, Tokyo, Japan). The optical textures were observed using a high-resolution polarizing microscope ECLIPS LV 100 with a Nikon LU Plan Fluor and Nikon CFI UW lenses without oil immersion (Nikon, Tokyo, Japan).

## 3. Synthesis

### 3.1. Synthesis of Dibromoazobenzene

**Scheme 2:**
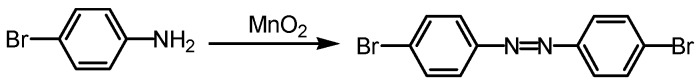
Synthesis of 4,4′-dibromoazobenzene [9].

As shown in Scheme 2, 4,4′-dibromoazobenzene was prepared from 4-bromoaniline through the oxidation of aromatic amines with active manganese dioxide (MnO_2_) [9]. This classic reaction, originally developed by Wheeler and Gonzales, is quite effective for synthesizing monomers to construct azobenzene-based conjugated polymers.

### 3.2. Synthesis of 4,4′-(4,4,5,5-tetramethyl-1,3-dioxaborolan-2-yl)azobenzene

**Scheme 3:**
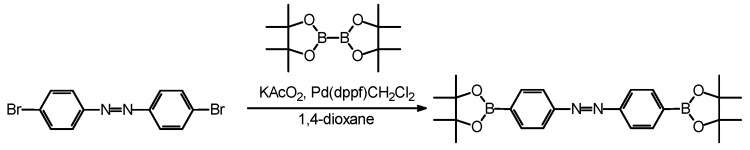
Synthesis of 4,4′-dibromoazobenzene. PdCl_2_(dppf)CH_2_Cl_2_: [1,1′-bis(diphenylphosphino)ferrocene]dichloropalladium(II)complex/dichloromethane. KAcO_2_: potassium acetate.

A mixture of bis(4,4,5,5-tetramethyl-[1,3]dioxolan-2-yl)borane and potassium acetate KAcO_2_ were dissolved in 1,4-dioxane. Next, [1,1′-bis(diphenylphosphino)ferrocene]dichloropalladium(II)complex/dichloromethane (PdCl_2_(dppf)CH_2_Cl_2_) was added as the catalyst to produce 4,4′-(4,4,5,5-tetramethyl-1,3-dioxaborolan-2-yl) azobenzene as a monomer (Scheme 3).

Under an argon atmosphere, 4,4′-dibromoazobenzene (0.503 g, 1.48 mmol), bis(4,4,5,5-tetramethyl-[1,3]dioxolan-2-yl)borane (0.753 g, 2.96 mmol), and potassium acetate (KAcO_2_, 0.593 g, 6.05 mmol) were dissolved in 1,4-dioxane (20mL) and added to an oven-dried Schlenk flask equipped with a stir-bar. Then, [1,1′-bis(diphenylphosphino)ferrocene]dichloropalladium(II)complex dissolved in dichloromethane (PdCl_2_(dppf)CH_2_Cl_2_, 0.065 g, 0.079 mmol) was added to the solution and stirred under reflux at 90 °C for 24 h. After cooling to room temperature, the reaction mixture was poured into water, extracted with dichloromethane, and washed with water. Further, the organic layer was dried over MgSO_4_ and filtered, and the solvent was evaporated. The product was purified by column chromatography (silica gel, eluent: hexane and ethyl acetate) to afford 0.450 g of the title compound (yield = 70%). ^1^H NMR (400 MHz; CDCl_3_; TMS) δ1.372 (s, 24H, C-C*H_3_*), 7.912 (d, 4H, 2,6,2′,6′-*H*(azobenzene), J = 6.4 Hz), 7.943 (d, 4H, 3,5,3′,5′-*H*(azobenzene), J = 6.4 Hz).

### 3.3. Polymerization

**Scheme 4:**
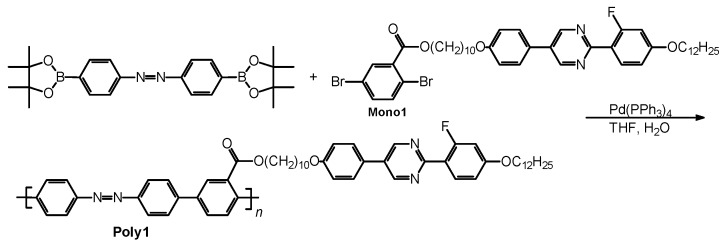
Synthesis of poly1.

Miyaura–Suzuki-type Pd(0)-catalyzed polycondensation was conducted between the azobenzene unit and the LC unit containing a mesogen ester (mono1) to yield poly1 (Scheme 4). Mono1 was previously prepared in our lab [15]. 

**Scheme 5:**
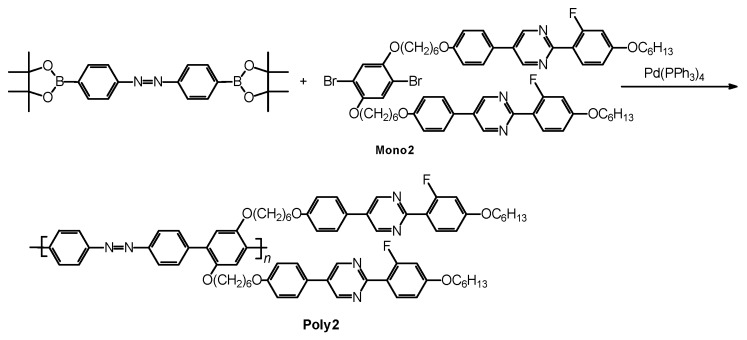
Synthesis of poly2.

As depicted in Scheme 5, the polycondensation reaction between the azobenzene unit and the disubstituted LC aromatic unit (mono2) was conducted using a Pd(0) catalyst to obtain poly2.

**Poly1**

Under an argon atmosphere, 4,4′-(4,4,5,5-tetramethyl-1,3-dioxaborolan-2-yl) azobenzene (40.0 mg, 0.0921 mmol) and the LC monomer (80.1 mg, 0.0942 mmol) were dissolved in THF (3.0 mL), added to an oven-dried Schlenk flask equipped with a stir-bar, and stirred for 0.5 h. Further, tetrakis(triphenylphosphine)palladium(0) (Pd(PPh_3_)_4_: 10.7 mg, 0.00926 mmol) was added to the solution, and potassium carbonate (K_2_CO_3_: 128 mg, 0.925 mmol) that was dissolved in water (1.0 mL) was separately added. The reaction mixture was stirred under reflux at 60 °C for 48 h. After cooling to room temperature, the reaction mixture was dissolved using a minimal amount of THF. The polymer in the THF solution was added to a large volume of methanol (poor solvent), resulting in precipitation. The suspension was further washed using a large volume of methanol to remove the catalyst and low molecular weight fractions. Subsequently, the polymer was collected by centrifugal separation and dried under vacuum to afford the desired product (yield = 70%).

**Poly2**

Under an argon atmosphere, 4,4′-(4,4,5,5-tetramethyl-1,3-dioxaborolan-2-yl) azobenzene (28.9 mg, 0.0666 mmol) and mono1 (78.0 mg, 0.0666 mmol) dissolved in THF (3.0 mL) were added to an oven-dried Schlenk flask equipped with a stir-bar, and the resulting solution was allowed to be stirred for 0.5 h. Then, tetrakis(triphenylphosphine)palladium(0) (Pd(PPh_3_)_4_: 10.5 mg, 0.00666 mmol) was added to the solution, and potassium carbonate (K_2_CO_3_: 92.6 mg, 0.666 mmol) that was dissolved in water (1.0 mL) was separately added. Further, the reaction mixture was stirred under reflux at 60 °C for 48 h. After cooling to room temperature, the reaction mixture was dissolved in a minimal amount of THF. The polymer in the THF solution was added to a large volume of methanol (poor solvent), resulting in precipitation. The suspension was further washed with a large volume of methanol to remove the catalyst and low molecular weight fractions. The polymer was collected by centrifugal separation and dried under vacuum to afford the desired product (yield= 75%).

## 4. Characterization

### 4.1. Molecular Weight Determinations

**Figure 2:**
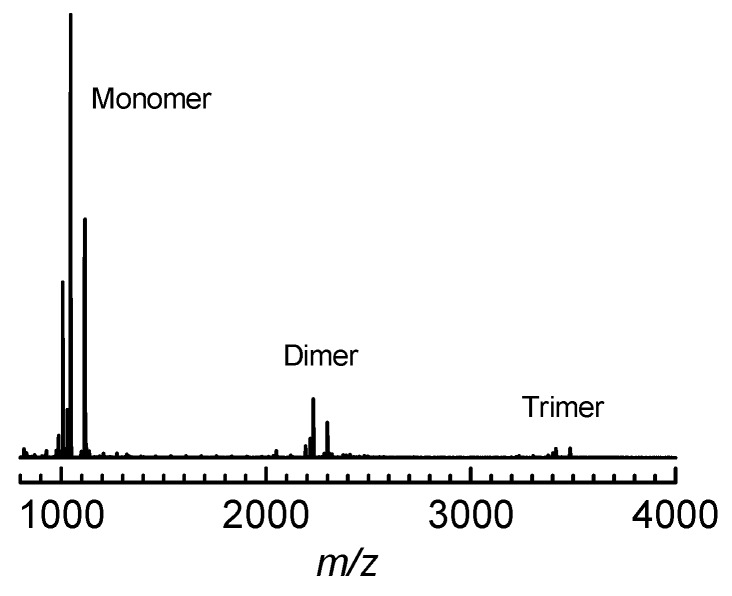
Matrix-assisted laser desorption/ionization time-of-flight mass spectrometry (MALDI-TOF MS) result for poly2.

GPC measurements were performed based on a polystyrene standard to determine the number- and weight-average molecular weights, i.e., (*M*_n_) and (*M*_w_), respectively, of the polymers (Table 1). The molecular weights were conveniently estimated based on the GPC experiments even though multi-angle light scattering detectors constitute a reliable technique. The *M*_w_ of poly1 was estimated to be 7900 g/mol with a dispersity value (*M*_w_/*M*_n_) of 1.15, whereas the *M*_w_ of poly2 was estimated to be 5300 g/mol with a dispersity value (*M*_w_/*M*_n_) of 1.06. The low molecular weight of the resulting polymers may be attributed to the large mesogens in the bulky monomer, which would decrease the activity of the Miyaura–Suzuki-type polycondensation. Further, poly1 and poly2 contained 8.9 and 4.5 monomer repeat units, respectively. The matrix-assisted laser desorption/ionization time-of-flight mass spectrometry (MALDI-TOF MS) of poly2 indicated monomer, dimer, and trimer as periodic signals, as shown in Figure 2. These results are indicative of the formation of the polymeric structure of poly2 by the polycondensation reaction.

### 4.2. IR Spectroscopy Measurements

**Figure 3:**
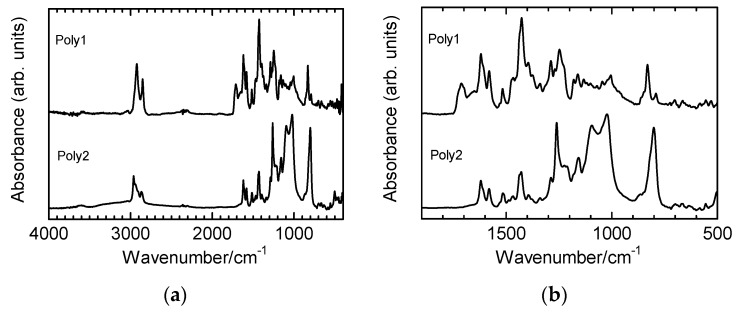
Infrared absorption (IR) spectra of poly1 and poly2 using the KBr method (**a**). IR absorption spectra of the fingerprint region (**b**).

FT–IR spectroscopy measurements were conducted to confirm the molecular structure of the polymers (Figure 3). The weak absorption peak at 3034 cm^−1^ was attributed to the C–H stretching of the aromatic protons, while the absorption band at 2950 cm^−1^ corresponded to the CH_2_ and CH_3_ stretching vibrations. The absorption band at 1710 cm^−1^ of poly1 was attributed to CO–O stretching vibration, which was not observed in the bands of poly2. The absorption peak at 1627 cm^−1^ corresponded to the C=C stretching of benzene, and the peak at 1580 cm^−^^1^ was likely due to stretching vibrations of N=N. The absorption bands attributed to ether stretching vibration were observed at 1245 cm^−1^ for poly1, and 1264 cm^−1^ for poly2. Finally, the Ar–H out-of-plane vibrations was observed at 829cm^−1^.

### 4.3. ^1^H NMR

**Figure 4:**
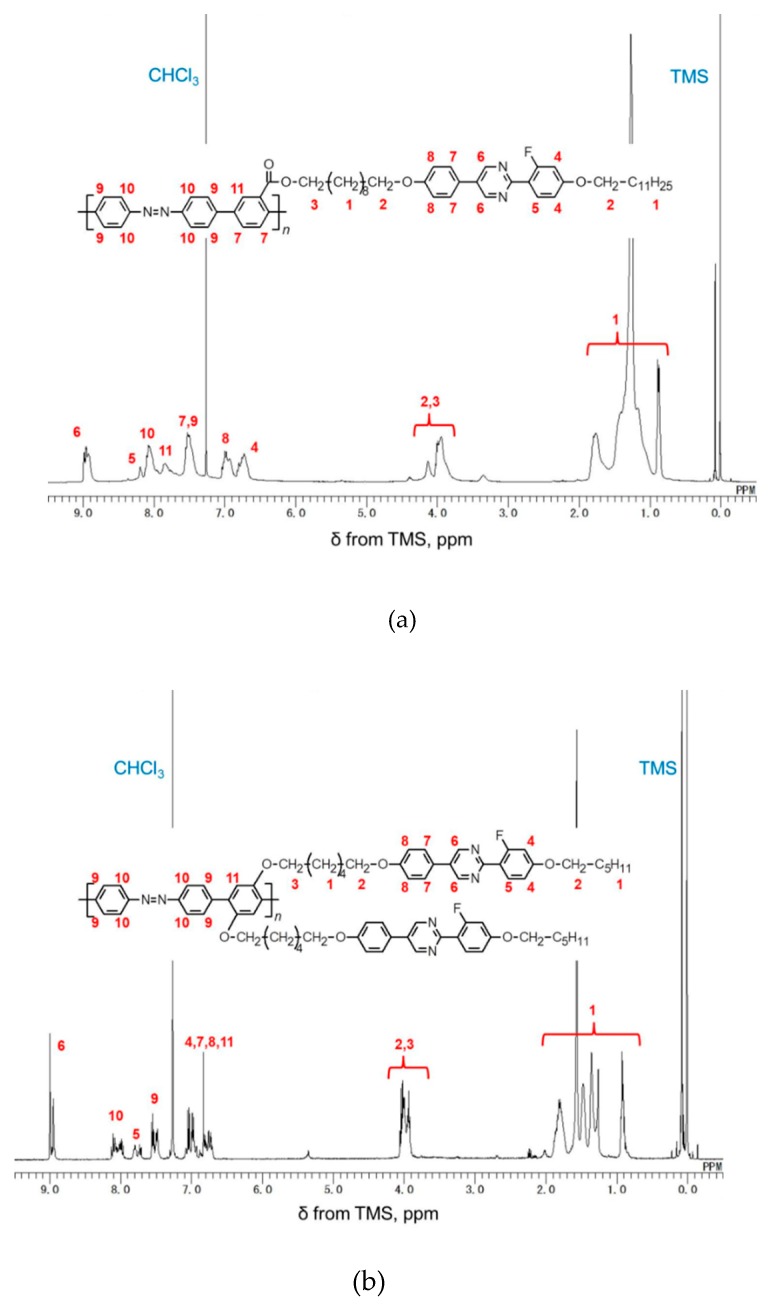
^1^H nuclear magnetic resonance (NMR) data of poly1 (**a**) and poly2 (**b**).

^1^H NMR (δ form TMS, tetramethylsilane, ppm) further confirmed the molecular structure of the polymers (Figure 4). The chemical structures of the polymers are confirmed.

### 4.4. UV-Vis and PL

**Figure 5:**
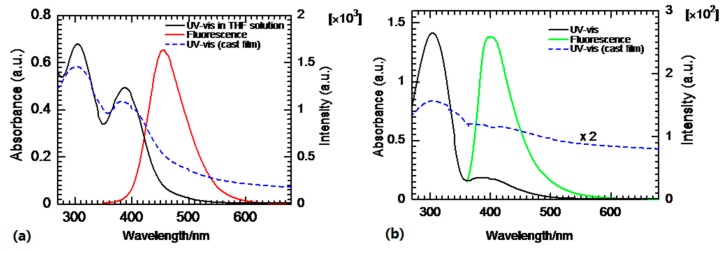
UV-vis absorption and fluorescent spectra of poly1 (**a**) and poly2 (**b**).

Figure 5 shows the ultraviolet-visible (UV-vis) optical absorption in THF solution and cast film (solid state after annealing with LC order) and the fluorescent spectra of poly1 and poly2 in THF solution. Poly1 exhibited absorption bands at 305 and 388 nm due to the π–π* transition of aromatic rings and π–π* transitions of the main chain, respectively. Poly2 exhibited these absorption bands at 303 and 383 nm. Poly1 and poly2 cast film in the form of LC order exhibited extended absorption toward long wavelengths (blue dashed lines in Figure 5). Poly1 emitted photoluminescence (PL) in THF solution at 455 nm (excitation wavelength: 340 nm), while poly2 emitted PL at 400 nm in the THF solution (excitation wavelength: 350 nm). The main chain of poly2 was considerably more twisted, resulting in a large dihedral angle between the monomer repeat units. This structural characteristic decreased the effective conjugated length of the polymer, resulting in a blue-shifted PL. 

### 4.5. Photoisomerization

**Figure 6:**
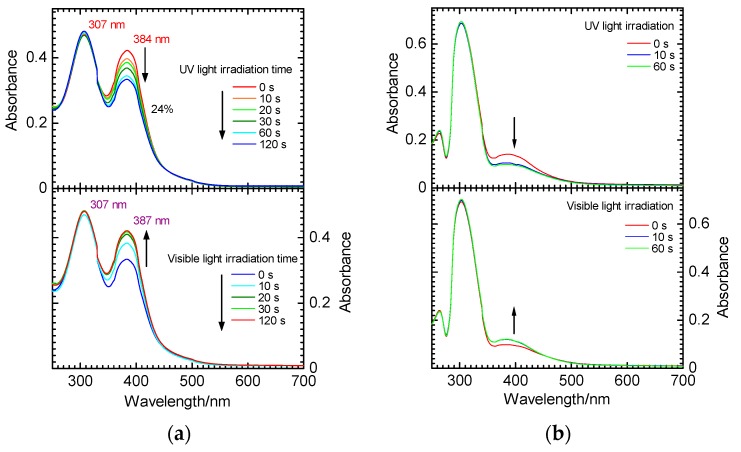
Change in the UV-vis absorption spectra upon irradiation with UV and visible light for poly1 and poly2 in tetrahydrofuran(THF) solution. (**a**) Poly1, (**b**) Poly2.

Figure 6 depicts the photoisomerization of the polymers in the THF solutions upon irradiation with UV and visible light. The irradiation of poly1 using UV light resulted in a decrease of the absorption band at 384 nm, which was accompanied by trans–cis photoisomerization. The absorption intensity of poly1 at 384 nm was decreased by 24% after irradiation with UV light for 120 s; however, irradiation using visible light restored the intensity of this absorption. By contrast, the optical response of poly2 under UV and visible light was considerably lower compared to that of poly1. The absorption peak at 307 nm in Figure 3 can be primarily attributed to the π–π* transition of the LC side chain. Further, the optical absorption of poly2 upon irradiation with visible light for 10 s and that with UV light for 60 s (Figure 6a, bottom) were found to overlap, which indicated that the cis–trans photoisomerization of poly2 in THF solution was completed after 10 s. 

### 4.6. Optical Texture

**Figure 7:**
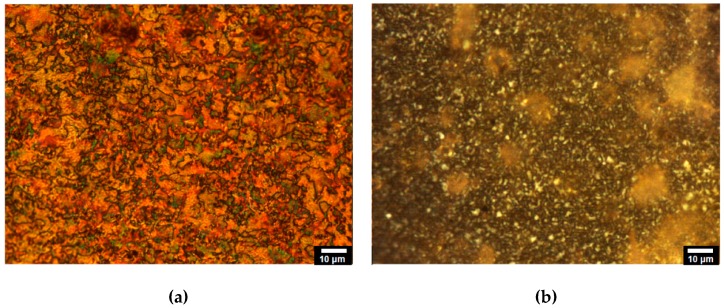
Polarizing optical microscopy images of poly1 (**a**) (at 200 °C, 500× and poly2 (**b**) (at rt, 1000×).

Figure 7 shows the polarizing optical microscopy (POM) images of poly1 and poly2. Poly1 displayed the Schlieren texture of the nematic phase, while poly2 exhibited a particle structure. However, definition of the typical feature (e.g., Schlieren texture, thread-like texture of nematic phase) of an LC optical structure could not be determined. Furthermore, the optical mesophase texture was maintained as a solid after cooling from the isotropic phase, and the texture did not change after further cooling.

### 4.7. Thermal Analysis

**Figure 8:**
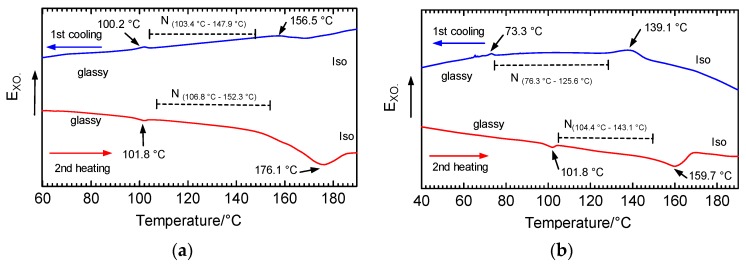
Dynamic scanning calorimetry (DSC) results for poly1 (**a**) and poly2 (**b**).

**Figure 9:**
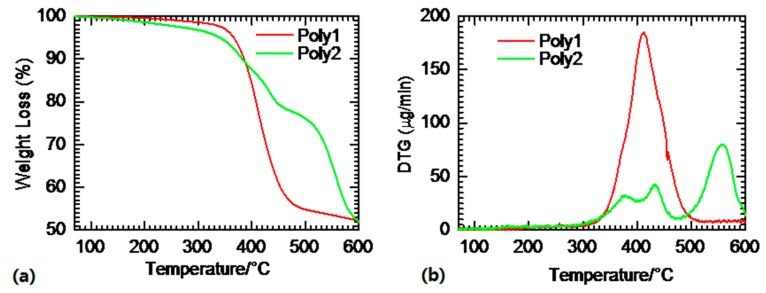
Thermogravimetry (TG) (**a**), and differential thermal analysis (DTA) (**b**) curves of poly1 and poly2.

Dynamic scanning calorimetry (DSC) curves for poly1 (Figure 8a) and poly2 (Figure 8b) show clear temperature ranges of the mesophase (LC phase) for these polymers. Figure 9 shows thermogravimetry (TG) and differential thermal analysis (DTA) of poly1 and poly2. For poly1, weight loss occurred at 410.3 °C, while poly2 experienced thermal degradation at 378.7, 433.9, and 555.3 °C. Decomposition and carbonization occurred at these temperatures. These results revealed that the polymers were in a stable LC phase at temperatures <278 °C. 

### 4.8. Synchrotron Radiation Grazing Incidence X-ray Diffraction (GI–XRD) 

**Figure 10:**
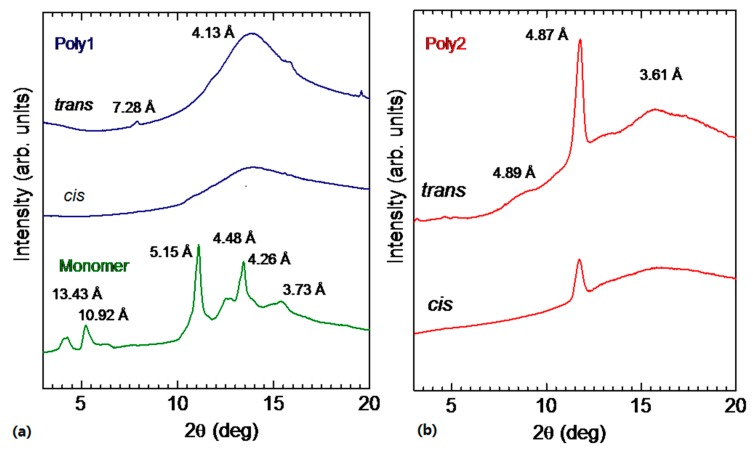
Synchrotron radiation grazing incidence X-ray diffraction (GI–XRD) analyses. (**a**) Poly1. (**b**) Poly2.

Synchrotron radiation grazing incidence X-ray diffraction (GI–XRD) analysis was performed to evaluate the microstructure of the polymers (Figure 10). The monomer exhibited signals that corresponded to the formation of the crystal structure. The cis form of poly1 prepared using UV light irradiation displayed a broad hollow diffraction at 4.13 Å. The signal at 7.28 Å was indicative of the trans isomer in poly1. While the cis form bending of the main chain could break the LC order, cis poly1 still showed a broad hollow at 4.13 Å, which indicated that the cis isomer of the polymer partially formed an LC order.

**Figure 11:**
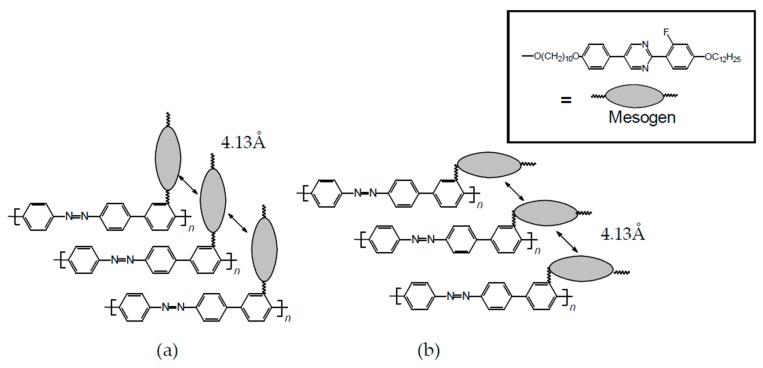
Possible structures of poly1. (**a**) Mesogens are positioned perpendicular to the main chain. (**b**) Mesogens are positioned parallel to the main chain.

**Figure 12:**
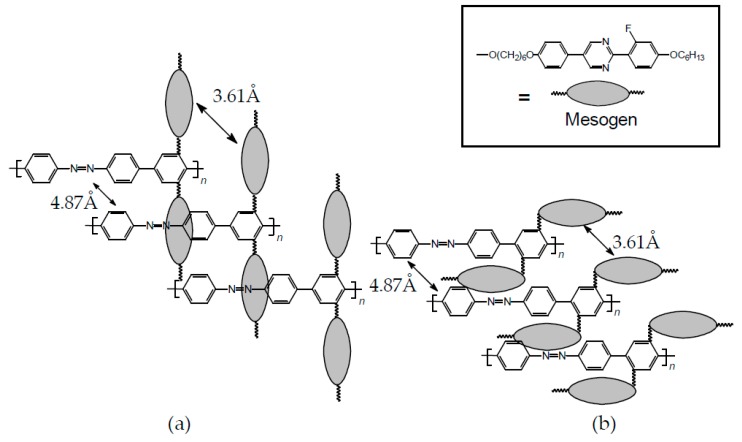
Possible structures of poly2. (**a**) Mesogens are positioned perpendicular to the main chain. (**b**) Mesogens are positioned parallel to the main chain.

Figure 11 depicts a possible structure of poly1. The XRD result indicated that the intermolecular distance between the side chain mesogens was 4.13 Å. The side chains may be situated in a perpendicular (Figure 11a) or parallel (Figure 11b) direction with respect to the main chain. Poly2 denotes signals at 4.89 Å and 4.87 Å and a broad hollow at 3.61 Å, which indicated that poly2 had a higher order LC structure. The diffraction signal at 4.89 Å was indicative of the cis form. The XRD analytical results for poly2 indicated that the intermolecular distance between the side chain mesogens was 3.61 Å, and the intermolecular main chain distances were likely to be 4.87 Å. The side chain of poly2 may be situated perpendicular (Figure 12a) or parallel (Figure 12b) to the main chain. The GI–XRD results demonstrated that photoisomerization can change the crystallinity of the polymer. This observation is one of the first reported examples of a detected change in the crystallinity of the polymers upon light irradiation.

## 5. Discussion

A classical reaction was conducted with MnO_2_ to obtain dibromoazobenzene as a monomer for subsequent Pd(0)-catalyzed Miyaura–Suzuki polycondensation to prepare azobenzene-based main chain-type conjugated polymers. The middle molecular weight polymer that was obtained from this reaction exhibited sufficient polymeric behavior, during cooling, including main chain-type photo-induced isomerization and mesophase–solid transitions. While this polymer maintained a stable LC structure in the solid-film form at rt with no formation of a crystal structure, the bulky side chain decreased its reactivity for increasing molecular weight in the polymerization, meaning high molecular weight polymers having an azobenzene unit in the polymer backbone may not exhibit photoisomerization behavior. 

The prepared polymers comprised a photoisomerization portion and an LC generator unit. The main chain was linearly rigid in the trans isomer state, which was suitable for showing main chain-type LC behavior. Therefore, the trans isomer polymer may be a side chain–main chain cooperation-type LC polymer when the side chains were aligned parallel to the main chain. The trans isomer of the polymer was obtained by visible light irradiation or thermolysis. The π-conjugated system with azobenzene units exhibited optical emission behavior. Repeated irradiation by UV and visible light of the polymers tuned the reversibility of the cis–trans isomerization. The LC disubstituted polymer (poly2) exhibited a low optical response compared with that of the mono-substituted polymer (poly1). This observation can be attributed to the mechanical wrapping of the main chain by the large LC group to shield from the external light, resulting in the reduction of light energy to the main chain. The large substituents likely restricted the mechanical motion of the main chain in the solid-state cis–trans photoisomerization reaction. POM analysis of the polymers confirmed the mesophase state. The mono-substituted polymer (poly1) exhibited a nematic phase, and the disubstituted polymer (poly2) contained no diffraction peaks at small angles in the XRD results, which indicated that poly2 formed a nematic phase without a layered structure; however, a sharp signal at the wide-angle region observed in the XRD results indicated the existence of a higher order structure. 

**Figure 13:**
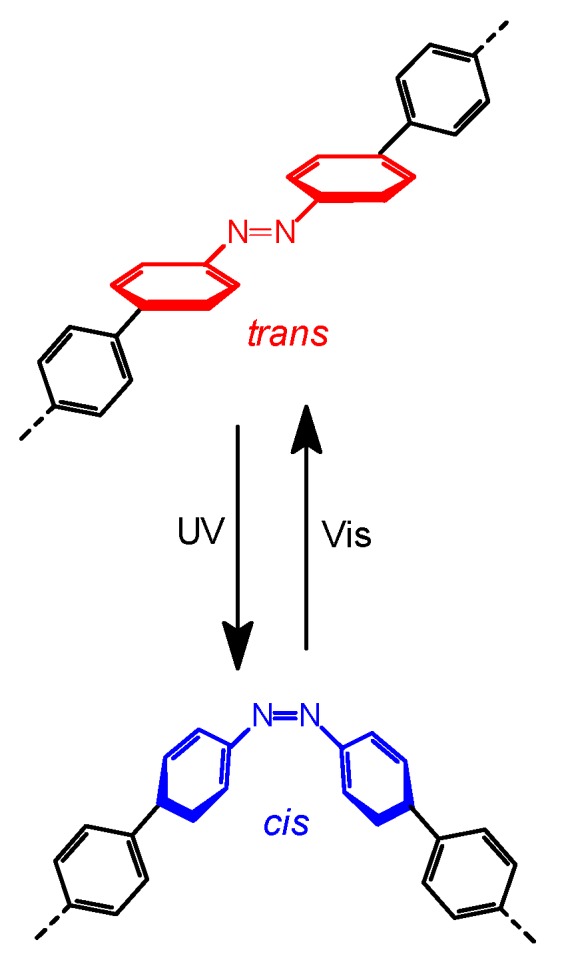
Plausible photoisomerization of the present polymer upon irradiation with UV and visible light.

The LC order was maintained after photoisomerization in the solid-film state. Figure 13 depicts a plausible structure of poly1 in cis and trans forms upon photoisomerization. The cis form exhibited lower crystallinity than the trans form. In addition, main chain isomerization could not be completed upon light irradiation because the residual trans isomer was observed after UV light irradiation, which may support the maintenance of the LC order. The trans form of the polymer was stable in the LC temperature range because of its high viscosity derived from the main chain type π-conjugated skeleton, which restricted photoisomerization and photo-induced phase transitions. Side chain-type non-π-conjugated polymers bearing azobenzene units have reportedly exhibited favorable photo-induced phase transitions [16,17].

## 6. Conclusions

Azobenzene are prevalent in chemistry, physics, and industrial applications. Therefore, developing new methods of preparing azobenzenes with desirable features is of particular interest. Thus, main chain-type polyazobenzenes exhibiting thermotropic liquid crystallinity behavior were prepared in this study. The photoisomerization reactions of the polymers were evaluated based on in situ UV-vis optical absorption spectroscopy measurements and GI–XRD results. This study resulted in the development of a convenient synthetic method for the further development of the azo-based polymer dye systems.

## Figures and Tables

**Table 1:** Molecular weights of the polymers ^a^.

Product	*M*_n_(g/mol)	*M*_w_(g/mol)	*M*_w_/*M*_n_	Number of Monomer Repeat Units ^b^
Poly1	6800	7900	1.15	8.9
Poly2	5000	5300	1.06	4.5

^a^ Molecular weights were estimated based on a polystyrene standard; ^b^ *M*_n_/[mass of monomer unit].

## References

[1] Bach H.C. (1966). Oxidative coupling of primary aromoatic diamines-aromatic azopolymers. ACS Polym. Prep..

[2] Kovacic P., Kyriakis A. (1963). Polymerization of benzene to p-polyphenyl by aluminum chloride-cupric chloride. J. Am Chem Soc..

[3] Berlin A.A., Liogon’KIĬ V.I., Parini V.P. (1961). Synthesis and properties of some aromoatic polymers. J. Polym. Sci..

[4] Gibbons W.M., Shannon P.J., Sun S.T., Swetlin B.J. (1991). Surface-mediated alignment of nematic liquid crystals with polarized laser light. Nature.

[5] Ichimura K., Suzuki Y., Seki T., Hosoki A., Aoki K. (1988). Reversible change in alignment mode of nematic liquid crystals regulated photochemically by command surfaces modified with an azobenzene monolayer. Langmuir.

[6] Shishido A., Shih M.Y., Khoo I.C. (2000). All-optical polarization holography by means of nematic liquid crystal doped with azobenzene liquid crystal. Proc. SPIE.

[7] Khoo I.C., Shih M.Y., Shishido A., Chen P.H., Wood M.V. (2001). Liquid crystal photorefractivity—Towards supra-optical nonlinearity. Opt. Mater..

[8] Ito S., Yamashita A., Akiyama H., Kihara H., Yoshida M. (2018). Azobenzene-based (meth)acrylates: Controlled radical polymerization, photoresponsive solid–liquid phase transition behavior, and application to reworkable adhesives. Macromolecules.

[9] Dai Y., Sun H., Pal S., Zhang Y., Park S., Kabb C.P., Wei W.D., Sumerlin B.S. (2017). Near-IR-induced dissociation of thermally-sensitive star polymers. Chem. Sci..

[10] Sun H., Dobbins D.J., Dai Y., Kabb C.P., Wu S., Alfurhood J.A., Rinaldi C., Sumerlin B.S. (2016). Radical departure: Thermally-triggered degradation of azo-containing poly(β-thioester)s. ACS Macro Lett..

[11] Huang S., Chen Y., Ma S., Yu H. (2018). Hierarchical self-assembly in liquid-crystalline block copolymers enabled by chirality transfer. Angew. Chem. Int. Ed..

[12] Hu J., Li X., Ni Y., Ma S., Yu H. (2018). A programmable and biomimetic photo-actuator: A composite of a photo-liquefiable azobenzene derivative and commercial plastic film. J. Mater. Chem. C.

[13] Cheng Z., Ma S., Zhang Y., Huang S., Chen Y., Yu H. (2017). Photomechanical motion of liquid-crystalline fibers bending away from a light source. Macromolecules.

[14] Wheeler O.H., Gonzalez D. (1964). Oxidation of primary aromatic amines with manganese dioxide. Tetrahedron.

[15] Kawabata K., Goto H. (2009). Liquid Crystalline π-Conjugated Copolymers Bearing a Pyrimidine Type Mesogenic Group. Materials.

[16] Yu H. (2014). Photoresponsive liquid crystalline block copolymers: From photonics to nanotechnology. Prog. Polym. Sci..

[17] Yu H. (2014). Recent advances in photoresponsive liquid-crystalline polymers containing azobenzene chromophores. J. Mater. Chem. C.

